# Non-Invasive Functional-Brain-Imaging with an OPM-based Magnetoencephalography System

**DOI:** 10.1371/journal.pone.0227684

**Published:** 2020-01-24

**Authors:** Amir Borna, Tony R. Carter, Anthony P. Colombo, Yuan-Yu Jau, Jim McKay, Michael Weisend, Samu Taulu, Julia M. Stephen, Peter D. D. Schwindt

**Affiliations:** 1 Sandia National Laboratories, Albuquerque, NM, United States of America; 2 Candoo Systems Inc., Coquitlam, BC, Canada; 3 StimScience, Inc., Berkeley, CA, United States of America; 4 University of Washington Seattle, Seattle, WA, United States of America; 5 The Mind Research Network and Lovelace Biomedical and Environmental Research Institute, Albuquerque, NM, United States of America; Boston Children's Hospital / Harvard Medical School, UNITED STATES

## Abstract

A non-invasive functional-brain-imaging system based on optically-pumped-magnetometers (OPM) is presented. The OPM-based magnetoencephalography (MEG) system features 20 OPM channels conforming to the subject’s scalp. We have conducted two MEG experiments on three subjects: assessment of somatosensory evoked magnetic field (SEF) and auditory evoked magnetic field (AEF) using our OPM-based MEG system and a commercial MEG system based on superconducting quantum interference devices (SQUIDs). We cross validated the robustness of our system by calculating the distance between the location of the equivalent current dipole (ECD) yielded by our OPM-based MEG system and the ECD location calculated by the commercial SQUID-based MEG system. We achieved sub-centimeter accuracy for both SEF and AEF responses in all three subjects. Due to the proximity (12 mm) of the OPM channels to the scalp, it is anticipated that future OPM-based MEG systems will offer enhanced spatial resolution as they will capture finer spatial features compared to traditional MEG systems employing SQUIDs.

## Introduction

Direct, non-invasive measurement of the human brain relies on sensing either the electric potential, electroencephalography (EEG), or magnetic field, magnetoencephalography (MEG) [1], or a combination of both [2]. These electromagnetic fields are mainly caused by neuronal current sources in the cerebral cortex [1, 3], and finding the precise location, orientation, and strength of these neuronal current sources with high spatiotemporal resolution remains a high priority for non-invasive functional brain imaging. While EEG methods benefit from the simplicity of the instrumentation, they suffer from significant setup time and low spatial resolution (~2 cm); this limitation is imposed by the neuronal return currents passing through the skull tissues, which have a low-conductivity profile compared to the surrounding cortex, dura, scalp, and skin tissues. MEG requires sophisticated instrumentation and measurement methods due to the extremely weak magnetic fields generated by the brain [1]. However, magnetoencephalography has superior spatiotemporal resolution to that of EEG, and its precision has continued to improve with advancing analysis methods [4, 5], instrumentation [6–11], and systems [12–16].

Traditional instrumentation for MEG measurements has been based on superconducting quantum interference device (SQUID) magnetometers, which employ a macroscopic quantum phenomenon using Josephson junctions [17]. While SQUID systems benefit from mature technology and analysis methods, they face two limiting factors: 1) fixed sensor positions, and 2) high maintenance cost. SQUID sensors operate at ~ 4 K and liquid helium is used to achieve cryogenic temperature. Regular costly maintenance is required to fill the helium reservoir and calibrate the SQUID-based MEG system, although recent advancements in helium recycling [18] is reducing maintenance costs. The main limitation of SQUID-based MEG systems, which also stems from the use of cryogens, is the fixed sensor position. Due to the use of liquid helium, a rigid Dewar is required to isolate the superconducting sensors from room temperature, hence the fixed position of sensors inside the rigid helmet. The requirement for efficient thermal insulation between the cold SQUID sensors and the subject’s head unavoidably increases the distance from the neural sources to the MEG sensors compared to having the sensors directly on the scalp. In commercial SQUID based MEG systems, the rigid helmet is designed to fit the 95th percentile male head size; this fixed helmet size increases the distance from the neuronal magnetic source to the senor and degrades the signal quality for individuals with smaller heads, e.g. children [18].

Optically pumped magnetometers (OPMs) offer a new paradigm for MEG measurements [6–11]. While the basic techniques for high sensitivity OPMs were demonstrated in 1969 [19], it took until the early 2000s to realize sub-femtoTesla sensitivity, a sensitivity rivaling that of the SQUID [7]. For this OPM, its sensing mechanism typically happens above room temperature and hence does not require cryogenic cooling. Each of the array’s sensors can be placed conformal to the individual subject’s scalp, minimizing the source-to-sensor distance and thus maximizing the signal strength.

In this paper, we report on the development of a 20-channel OPM-based magnetoencephalography system to study complex neural circuits, non-invasively, in human subjects. Our instrumentation and data analysis pipeline are improved over our prior work presented in [14]. We have added source localization capability to our MEG system and have modified our instrumentation to enhance its source localization accuracy. Employing the upgraded system, we conducted new MEG experiments. To validate the functionality of our MEG system, we used a 306-channel Elekta-Neuromag SQUID-based MEG system as our “gold standard” and localized somatosensory evoked magnetic fields (SEF) and auditory evoked magnetic fields (AEF) in three adult subjects with sub-centimeter accuracy.

## Materials and methods

### Magnetic sensor

**Fig 1:**
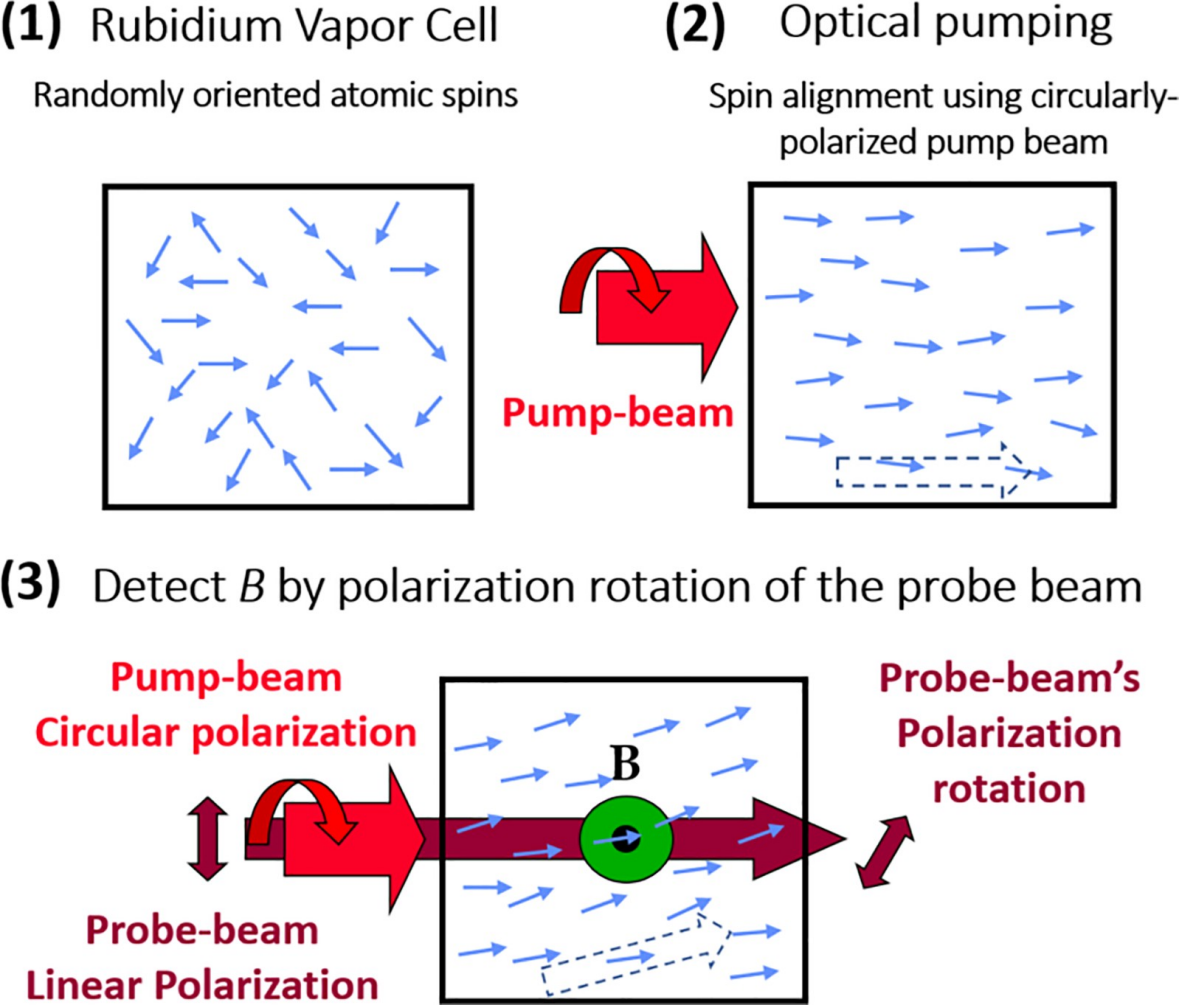
Optically-pumped-magnetometer’s principle of operation. (1) The rubidium atoms of the vapor cell have randomly oriented atomic spins. (2) Using the circularly polarized pump-laser (795 nm), the spins are oriented in the propagation direction of the pump-laser, generating a macroscopic magnetization of Rb vapor (dotted arrow). (3) Due to an external magnetic field (**B**) the atomic spins precess, causing the magnetization vector of the ensemble of atoms to have a constant angle relative to the pump beam axis approximately proportional to **B**. Through rotation of the probe beam polarization due to the Faraday effect, one can deduce the component of magnetization along the pump/probe beam axis.

The principle of operation of low-field optically pumped magnetometry is illustrated in Fig 1. These sensors operate in the spin-exchange-relaxation-free (SERF) regime [7]. At the heart of these optically pumped magnetometers is a vapor cell where the light-atom interaction takes place. The vapor cells in our system contain a small droplet of rubidium (^87^Rb) and by heating up the vapor cells to a temperature of ~ 150°C, we achieve a high density of rubidium atoms (~10^14^ cm^-3^). A circularly polarized pump-laser, tuned to rubidium’s D1 spectral resonance line (795 nm), orients the atomic spins, magnetizing the atomic vapor. The rubidium atoms precess in the presence of an external magnetic field, and the presence of a small magnetic field induces an angle in the magnetization relative to the pump axis. The magnetization of the atoms induces Faraday rotation in the linearly polarized probe beam (780 nm) [20]. The probe beam is tuned near to the D2 resonance of Rb and propagates colinearly with the pump beam. The polarization rotation angle due to the Faraday effect is approximately proportional to the component of magnetization along the pump/probe beam axis and thus gives us a mechanism with which to measure the magnetic field [8].

**Fig 2:**
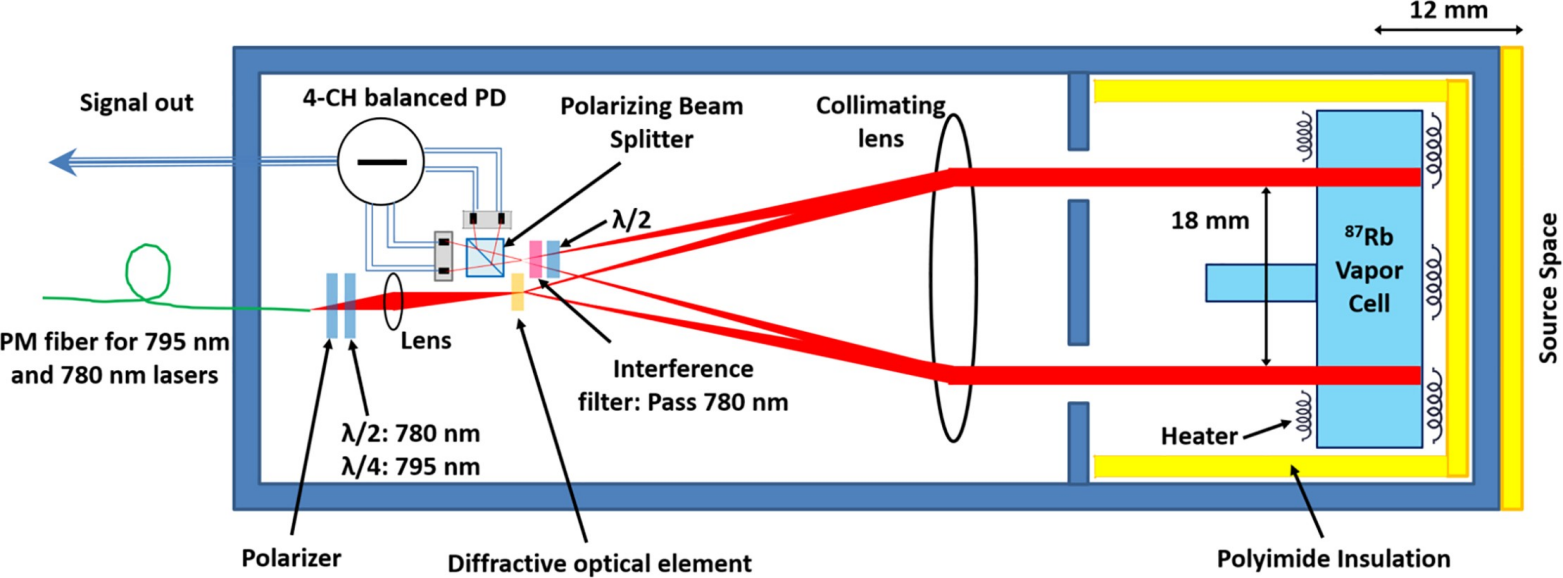
The OPM sensor’s schematic [8]. PBS: polarizing beam splitter; PM: polarization maintaining, PD: photodiode, λ/2: half wave plate, λ/4: quarter wave plate.

The magnetic sensor of our system is the OPM described in [8] (Fig 2). Our sensor features 4-channels formed at the intersection of four laser beams with the vapor cell’s rubidium atoms. The four laser beams have a separation of 18 mm with a full width at half maximum (FWHM) of 2.5 mm. Based on the optical path length of the vapor cell, 4 mm, each channel has a sensing volume of $4\;mm\times \pi \times {\left(\frac{FWHM}{2}\right)}^{2}=20\;{mm}^{3}$. To achieve thermal isolation between the vapor cell walls and the subject’s scalp, we have added an extra layer of polyimide insulation with a thickness of 3 mm. Hence, the total distance between the subject’s scalp and the geometric center of the channels sensing volume is 12 mm. Using on-sensor coils, a 1 kHz modulating magnetic field is applied to each sensor’s vapor cell in either a vertical or horizontal direction, referenced to the sensor’s frame of reference, and perpendicular to the direction of the laser beams. The modulating magnetic field defines the sensing axis for the sensors. Thus, each magnetometer channel measures a component of the magnetic field that is transverse to the scalp, and the measured component can be switched by changing the direction of the modulation.

### System components

**Fig 3:**
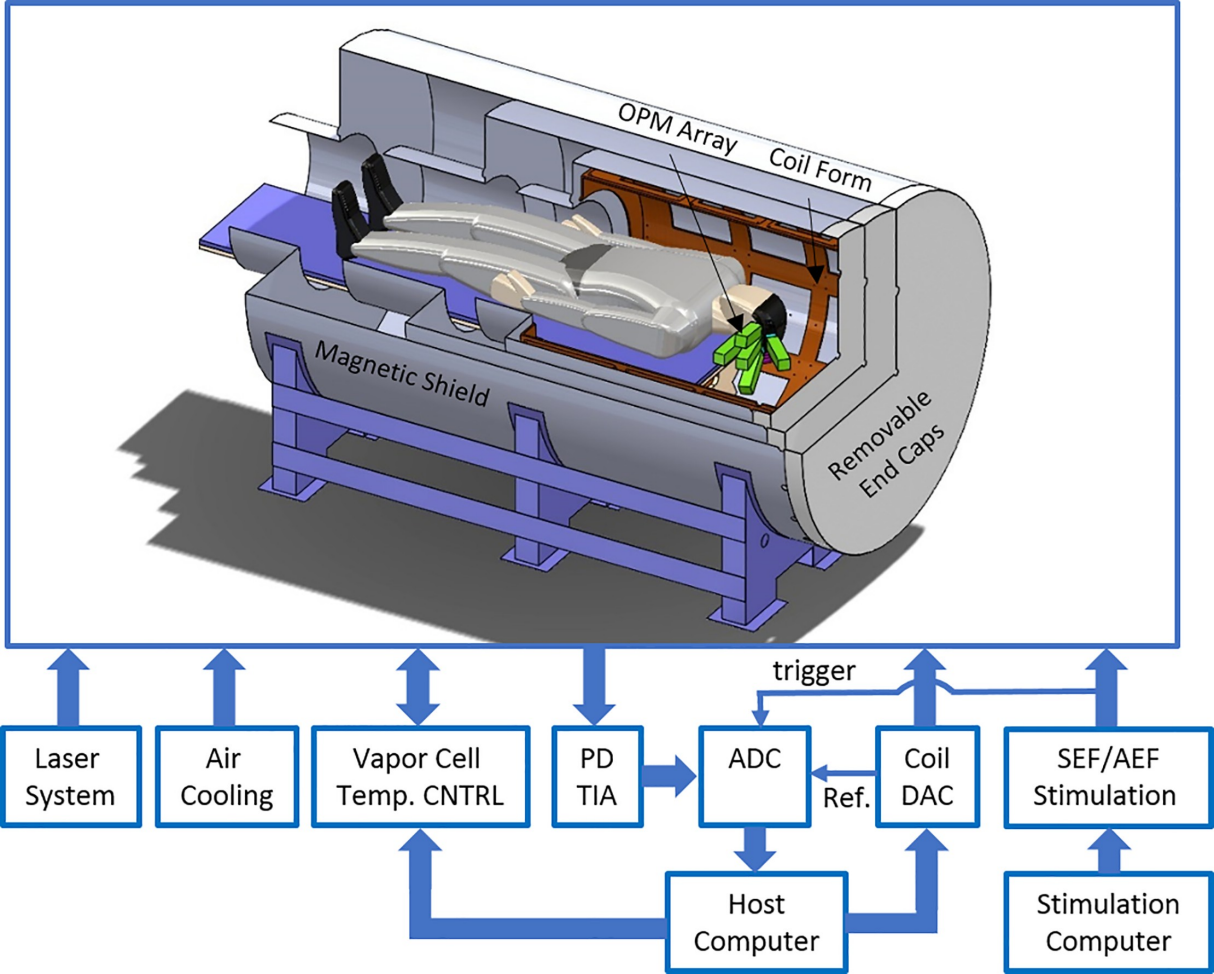
The MEG system block diagram [14]. PD TIA: the transimpedance amplifier which amplifies the currents from the sensors’ photo diodes; temp. CNTRL: temperature control; ADC: analog-to-digital converter; DAC: digital-to-analog converter; SEF/AEF Stimulation: somatosensory/auditory stimulation; Ref.: 1 kHz reference for the software lock-in amplifier.

In [14], we discuss in detail the design and characterization of our OPM-based MEG system. The system block diagram including a drawing of the magnetic shield containing the subject and OPM array is shown in Fig 3. Multiple subsystems were developed: 1) five 4-channel OPMs; 2) a person-sized magnetic shield with magnetic field control; the shield has a length of 269 cm and an external diameter of 140 cm. There are 18 coils embedded in the shield to null the remnant DC magnetic field around the sensor array; 3) a laser and optical system to provide light to the five sensors; 4) a data acquisition (DAQ) system to digitize the sensors’ signals; 5) custom-electronics for closed-loop vapor cell temperature control; 6) custom-electronics to operate the sensors and null the remnant DC magnetic field inside the shield; and 7) customized LabVIEW (National Instruments, US) software for data acquisition and system control.

### Signal path

**Fig 4:**
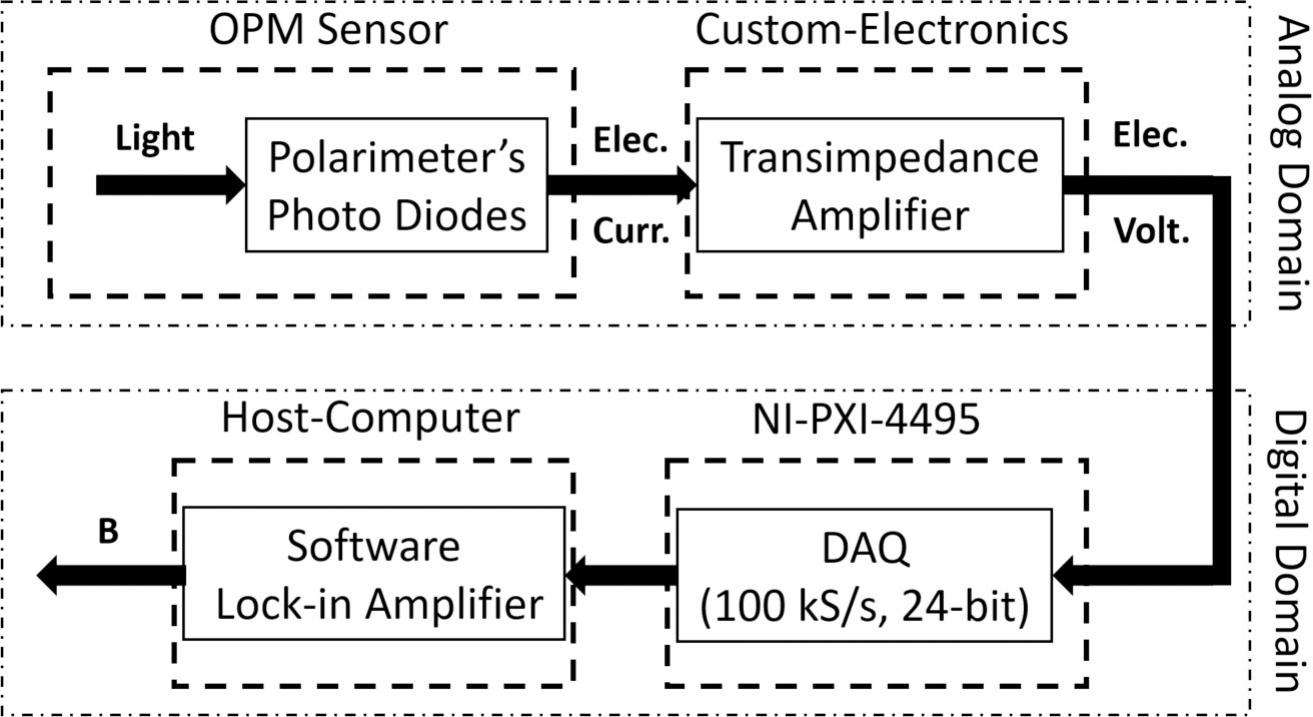
The MEG system’s signal path: the probe beam’s polarization is converted into electrical current by the sensor’s polarimeter; the amplified electrical current is digitized by a sampling rate of 100 kS/s and a resolution of 24-bit using commercial data acquisition cards; using a custom-designed software lock-in amplifier (LIA) the sensed magnetic flux density is calculated and stored on the host computer’s hard drive.

The signal path’s block diagram is depicted in Fig 4. The electric current from the sensor’s photodiodes is sent to a transimpedance amplifier (TIA) (QuSpin, Inc.) and the resulting output voltage is digitized by the MEG system’s data acquisition module at 100 kS/s with 24-bit resolution. The digitized raw signal carries a 1 kHz modulation due to the applied field modulation. The AC amplitude and phase of this light polarization rotation signal is proportional to the magnetic field sensed by the OPM. A software lock-in amplifier (LIA) implemented in LabVIEW demodulates the sensor signal and stores the digitized data to the hard drive at a decimated rate of 1 kS/s.

## Methods

### Experimental setup

For conducting OPM-MEG measurements, the subject lies in the supine position inside the custom-designed magnetic shield. During the OPM-MEG experiment, the 20-channel OPM array covers the left hemisphere of the subject’s head. Depending on the type of MEG experiment, i.e. AEF vs. SEF, the subject is guided to tilt his/her head such that the OPM array covers either the auditory or somatosensory cortices.

We conducted the MEG experiments on three healthy male subjects aged between 38 and 43 years old. We defined the accuracy of our MEG system as the distance between the locations of the localized neuronal current sources yielded by our OPM-based MEG system and a commercial SQUID-based MEG system. For comparison purposes, the MEG experiments were conducted with the same protocols using a 306-channel Elekta-Neuromag SQUID system (Elekta, Sweden) located in a magnetically shielded room (MSR) at the Mind Research Network (Albuquerque, NM). The protocols of the MEG experiments were approved by the Human Studies Board (HSB) of Sandia National Laboratories and the Chesapeake Institutional Review Board (IRB). Prior to experiments, an informed written consent was obtained by the project’s primary investigator from all the participants.

The main advantage of an OPM-based MEG system over their SQUID counterpart is higher SNR due to smaller distance between the sensors and the subject’s scalp which stems from the room temperature operation of these devices. To capitalize on this advantage, OPM sensors must be adjusted to conform to the subject’s scalp. In the presented work, the sensors are fixed in place, and the subject’s head is gently pushed against the array with an air bladder. The adjustment of the sensor positions over this limited area would result in changes of the sensor positions of ~1 mm from subject to subject.

To assess the functionality of the MEG-based OPM system, its source localization capabilities should be cross validated relative to commercial SQUID-based MEG systems. We apply an equivalent current dipole fitting algorithm to the processed brain signal; this algorithm assumes characteristics of the sensors, i.e. position, gain, and sense-angle, are constant during the whole MEG experiment. In this context sense-angle refers to the vector component of the magnetic field measured by the magnetometer with respect to a coordinate reference system. If channel position, gain, or sense-angle varies between trials, it can induce significant localization error [21]. We calibrate our OPM array sensors before each MEG experiment by measuring the channels’ gain and sense-angle; the sensors are assumed to be stationary during the experiment, and their spatial coordinates are extracted from mechanical drawings of the array holder and the sensors.

**Fig 5:**
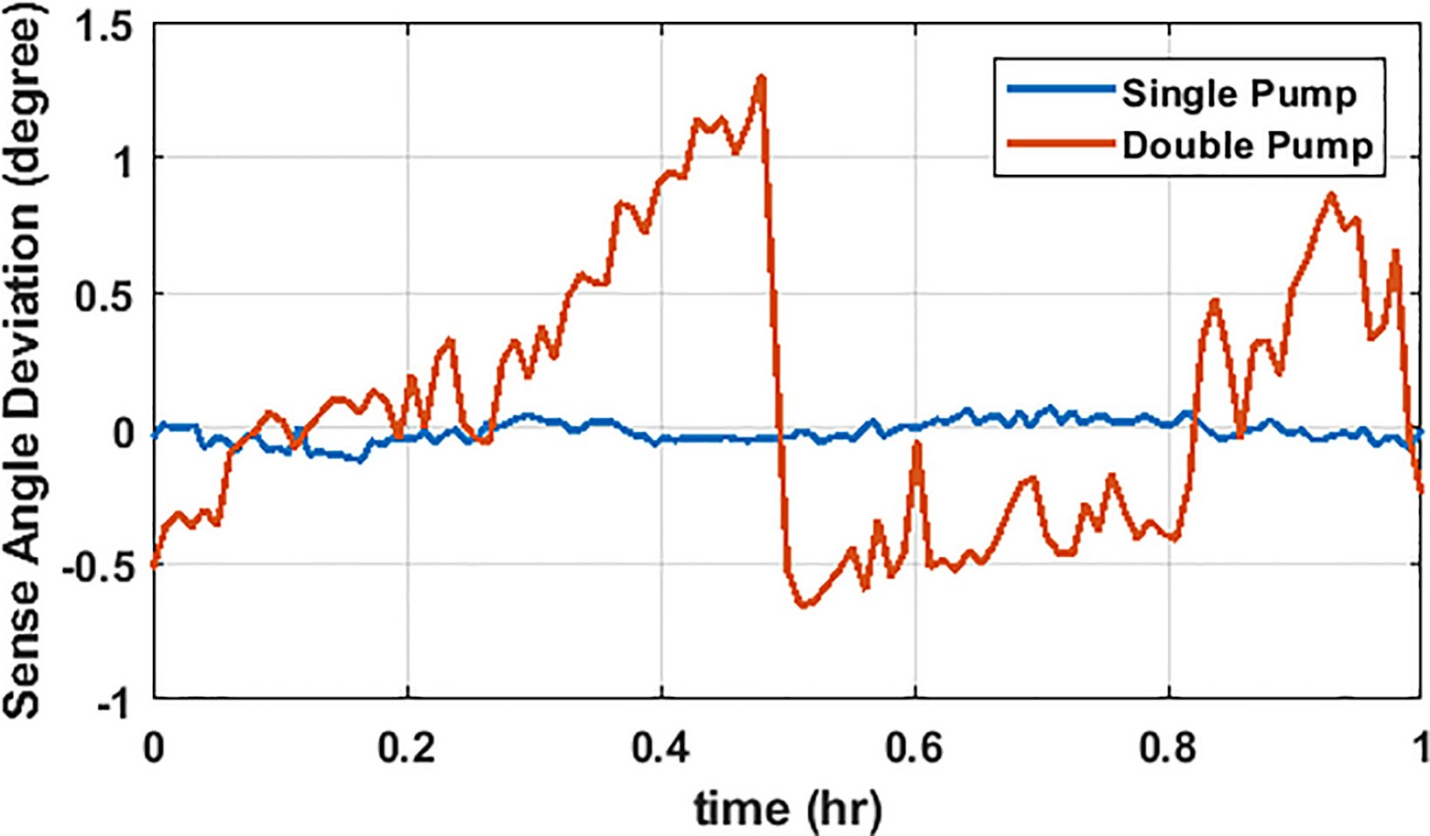
Stabilizing the gain and sense-angle. The sense-angle is measured over the course of two hours using single- and double-pump-laser systems. The single-pump-laser system has reduced the sense angle variation by more than an order of magnitude.

We observed gain fluctuation (~5%) and sense-angle variation (~ 2.5°) in our OPM sensors. The gain fluctuations of our OPM sensors are dominated by the probe-laser amplitude variation at the sensor level. Laser amplitude fluctuation can be minimized by a more stable mechanical design that limits drift in coupling to optical fibers or by adding active feedback. Ideally the sensor’s sense-angle is dependent only on the orientation of the on-sensor coils and their currents; hence, constant throughout the experiment. However, for the configuration of the sensors as implemented in [14], we observed a significant fluctuation in the sense-angle (~ 2.5°) over the course of a couple of hours (Fig 5); this fluctuation was attributed to variation in the amplitude of the two frequencies of light composing the pump-laser amplitude at the sensor level. This relative amplitude variation causes a varying light shift (AC stark shift), which produces a varying fictitious magnetic field along the direction of the pump laser [22]. A magnetic field (either light induced or real) along the pump laser direction causes the sense axis to rotate in the plane perpendicular to the pump axis [23]. Originally, the two frequencies of the pump laser were introduced to maximize the magnetic response of the OPM sensor. In this “double pump” scheme, the pump-laser was detuned by +10 GHz from the D1 optical transition in rubidium maximizing the OPM response [8]. To compensate for the intentionally introduced light shift [22], a second pump-laser with a detuning of -10 GHz is employed [8]. Due to imperfect alignment of the light polarization to the stress axis of the polarization maintaining fiber, particularly after passing through fiber splitters, the two pump-lasers were experiencing a different polarization rotation after propagating through the fiber and then a different amplitude after passing through the sensor’s polarizer; the different polarization rotation between the two colors of the pump laser is due to the slight difference of index of refraction of the fibers at the different frequencies of the pump, and the polarization rotation varies with the ambient temperature. The variation between the two pump-lasers’ power level in the sensor created the sense-angle fluctuations. By switching from a double-pump-laser scheme to a single-pump-laser scheme, we were able to reduce the sense-angle drift by an order of magnitude as shown in Fig 5. In the single-pump-laser-scheme, the pump is tuned to the D1 resonance to minimize the light shift. This hardware improvement was critical to enhance the localization accuracy of our MEG system in the subsequent MEG experiments.

#### Auditory Evoked Magnetic Fields (AEF)

To activate the auditory cortex of the subjects, they were presented with a series of standard 1 kHz tones and rare 1.2 kHz tones. The pulse duration of both tones was set at 100 ms and they were presented with random intervals around 1.1 s. Non-magnetic earphones (Etymotic Research, Inc., US) delivered the audio stimuli to the subject’s ears. The earphones receive the audio signal from the host computer’s audio card which is controlled by the stimulus delivery program Presentation (Neurobehavioral Systems, US). Apart from the audio card, the stimulus delivery program also controls the computer’s parallel port (LPT). The LPT port is used to send two trigger signals, associated with standard/rare tones, to the MEG system’s data acquisition module. Synchronization between the presented audio signals and the trigger signals was measured with a jitter of < 200 μs. The subject is presented with a total of 360/150 audio pulses for the standard/rare tones.

#### Somatosensory Evoked Magnetic Fields (SEF)

To stimulate the somatosensory cortex of the subjects, we sent current pulses to the subject’s median nerve on the right wrist through two 8-mm felt pads, spaced 25 mm apart. The unipolar stimulus signal had a pulse-width of 200 μs, and its amplitude was set, individually for each subject, according to the 2-cm thumb twitch response. The stimulus delivery program, Presentation (Neurobehavioral Systems, US), running on the host computer, controlled the timing of current stimulus by sending a trigger signal through the computer’s parallel port (LPT) to the stimulator module. The stimulator module is a commercial constant-current high-voltage peripheral stimulator, DS7A (Digitimer, United Kingdom). The stimulator’s trigger is also routed to the MEG system’s data acquisition module which collects the MEG data simultaneously with the trigger signal. The stimulus delivery program sends 400 trigger pulses, with random intervals around 1 s, to the DS7A stimulator.

### Signal processing pipeline

The signal processing pipeline processes the stored magnetoencephalography data, constructs the forward model, and localizes the neuronal current sources. For MEG signal processing tasks, we use Fieldtrip [24], a Matlab (MathWorks, Inc.) toolbox offering advanced analysis methods for MEG.

#### Magnetoencephalography data processing

The digitized, time-domain magnetoencephalography data is bandpass filtered from 0.5–150 Hz. The power of the filtered signal is used as a criterion to detect corrupted channels. After removing the malfunctioning channels, showing poor noise performance, the segments of the continuous, bandpass-filtered signal, which are contaminated with 1) discontinuities, 2) muscle/movement artifacts, and 3) electrooculogram (EOG) artifacts, are identified and subsequently removed from the continuously recorded data. Using the trigger signals recorded alongside the MEG data, the trials are defined for the clean data segments. Depending on the subject’s movements during the experiment, this step might remove as many as half the trials. The remaining trials are averaged relative to the onset of the stimulus trigger.

Assuming neuronal current sources are statistically independent from noise sources such as 60 Hz, heart’s magnetic field, etc., independent component analysis (ICA) [25, 26] is used to decompose the recoded data into statistically independent sources. We employ the ICA algorithms embedded in Fieldtrip. The unmixing matrix yielded by the ICA algorithm, is used to remove the noise sources from the time-locked sensor-level channels.

#### Constructing the forward model

The forward model in the context of functional brain imaging calculates the sensor-level magnetic topography, emanating from a specific neuronal current source [3]. The first step in calculating the forward model is finding the position of the subject’s brain relative to the sensors, a procedure commonly referred to as the co-registration of magnetic resonance imaging (MRI) and MEG data. The basic assumption in MEG-MRI co-registration is that the subject’s brain does not shrink or move inside the skull between the MRI and MEG sessions.

Four head position indicator (HPI) coils were placed on the subject’s scalp prior to running the MEG experiment. The positions of the HPI coils along with the subject’s head shape were digitized using an electromagnetic tracking system: Fastrack digitizer (Polhemus, US). Before presenting the subject with stimuli, a sequence of sinusoidal currents with a peak-to-peak amplitude of 2 mA and a frequency of 5.1, 5.2, 5.3, and 5.4 Hz are applied sequentially to the HPI coils. The magnetic field emanating from these coils is used for the dipole fitting routine [27] embedded in the Fieldtrip, determining the HPI coils positions relative to the OPM array. The coordinates of the HPI coils are used for MEG-MRI co-registration.

The volume conduction model, i.e. head model, is calculated using the single-shell head model [28] provided in the Fieldtrip package. The head model estimates the return/volume current given a primary current dipole at a specific location inside the model volume; and in MEG both primary and return/volume currents contribute to the magnetic field outside the skull. The single-shell head model yields an accuracy comparable to that of the boundary element method (BEM) for MEG signals but avoids the tedious computations of BEM model [28].

#### Neuronal current source localization

The inverse problem in the context of functional brain imaging refers to finding the locations and orientations of neuronal current sources underlying a measured sensor-level magnetic spatial topography [1, 3]; this is an inherently ill-posed problem with multiple source solutions possible for a given magnetic topography [1, 3]. The precision of neuronal current source localization method depends on the quality of the sensor-level MEG data, stability of the magnetic sensors, and the robustness of the forward model. We apply an equivalent current dipole (ECD) fitting algorithm to localize neuronal current sources activated in the AEF and SEF experiments. Dipole fitting is an iterative algorithm which minimizes the error between model and measured magnetic field [1]. In this method, the (neuronal current) source is modeled as an equivalent current dipole; the neurophysiological reasoning behind this assumption is that activated pyramidal cells in the cortical regions are aligned side by side and the generated magnetic field is measured at a distance by the sensors [3] such that simultaneously active focal groups of neurons approximate a point source from the point of view of the sensors positioned outside of the head.

We used Fieldtrip to implement our dipole fitting routine. We defined the grid points across the whole brain, rather than selecting cortical regions. With a resolution of 0.5 cm, we complement our grid scanning procedure with non-linear search to fine-tune the position and orientation of an optimal single current dipole. For AEF, even though there are two simultaneously activated bilateral cortical regions, it is safe to assume that a single current dipole is an adequate source model; this assumption is based on the fact that our OPM sensor array covers the left hemisphere and, due to large distance, does not sense the magnetic field stemming from the activated auditory cortex in the right hemisphere. For SEF, we stimulate the median nerve of the right wrist, hence the early activated somatosensory cortex (N20m) is in the left hemisphere where the OPM sensor array is located.

## Results

### Array characterization

**Fig 6:**
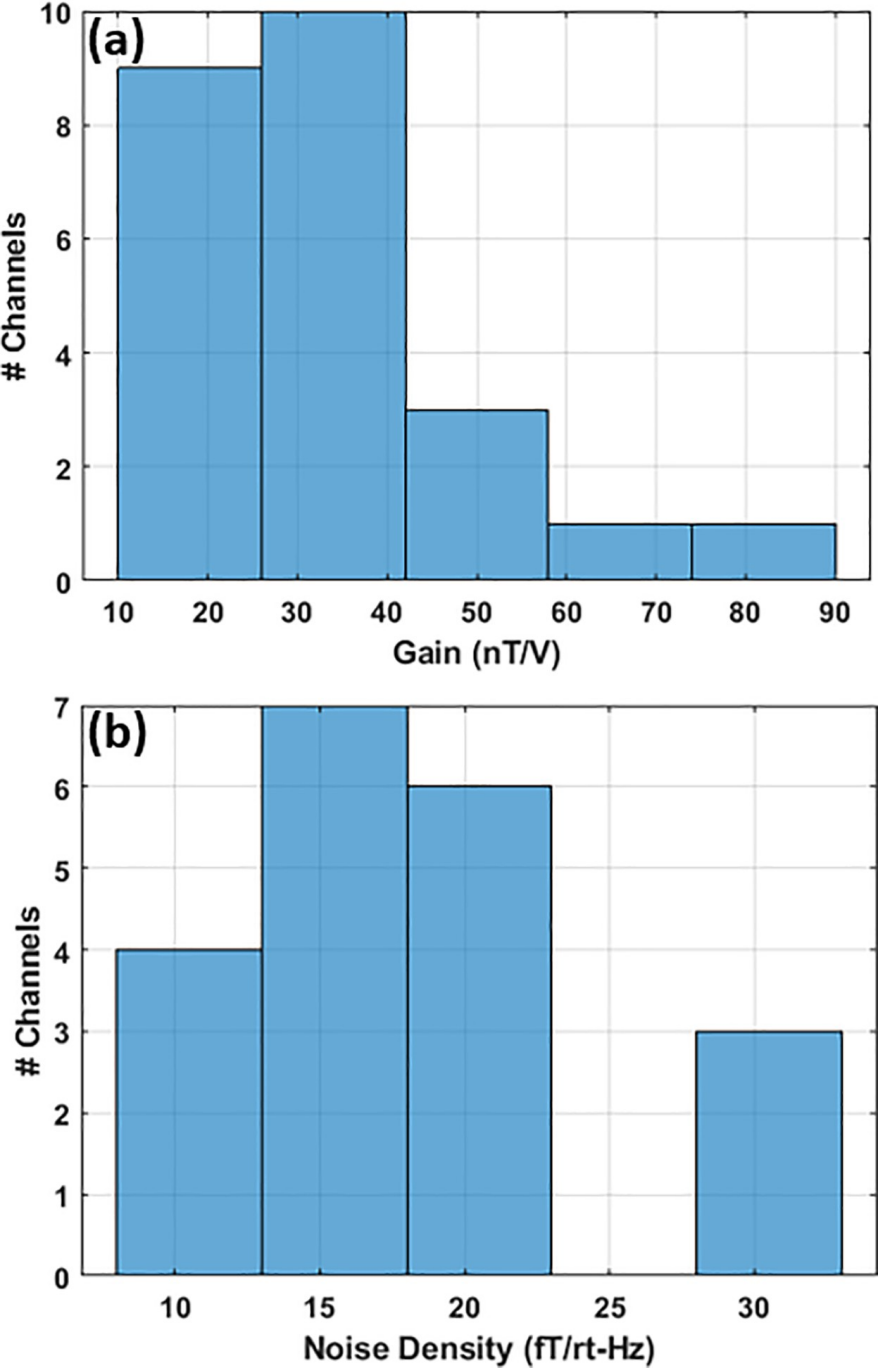
OPM array characterization: the histograms of (a) gain across channels, and (b) channels average noise density estimated between 10–44 Hz.

The person-sized magnetic shield, housing the sensor array, provides a measured shielding factor of ~1600 in the longitudinal direction. Using commercial fluxgates, the vectoral transfer function of the coils embedded in the shield, are measured at various locations in the vicinity of the sensor array. Using non-linear optimization, we solve for a set of DC current values for the 18 embedded coils to minimize the remnant DC magnetic field as is measured by the sensor array. By employing this technique, we have successfully reduced the background magnetic field to < 0.3 nT along the two sensitive axes of each sensor. Sensor conversion factors, i.e. gain, are measured using the shield coils, as shown in Fig 6-A. There are several factors affecting the measured gain including number of alkali atoms which is a function of temperature, and laser power; hence, the measured gain varies among the channels as depicted in the histogram of Fig 6-A. Fig 6-B shows the histogram of the measured noise performance. The average noise is estimated between 10–44 Hz after cleaning the array data using ICA. Channels with low conversion factor have poor noise performance; however, noise performance is impacted by other factors such as how well the polarimeter is balanced and cancels the laser amplitude noise, ambient magnetic noise, shield vibration/acoustic noise, etc.

### Forward model construction

**Fig 7:**
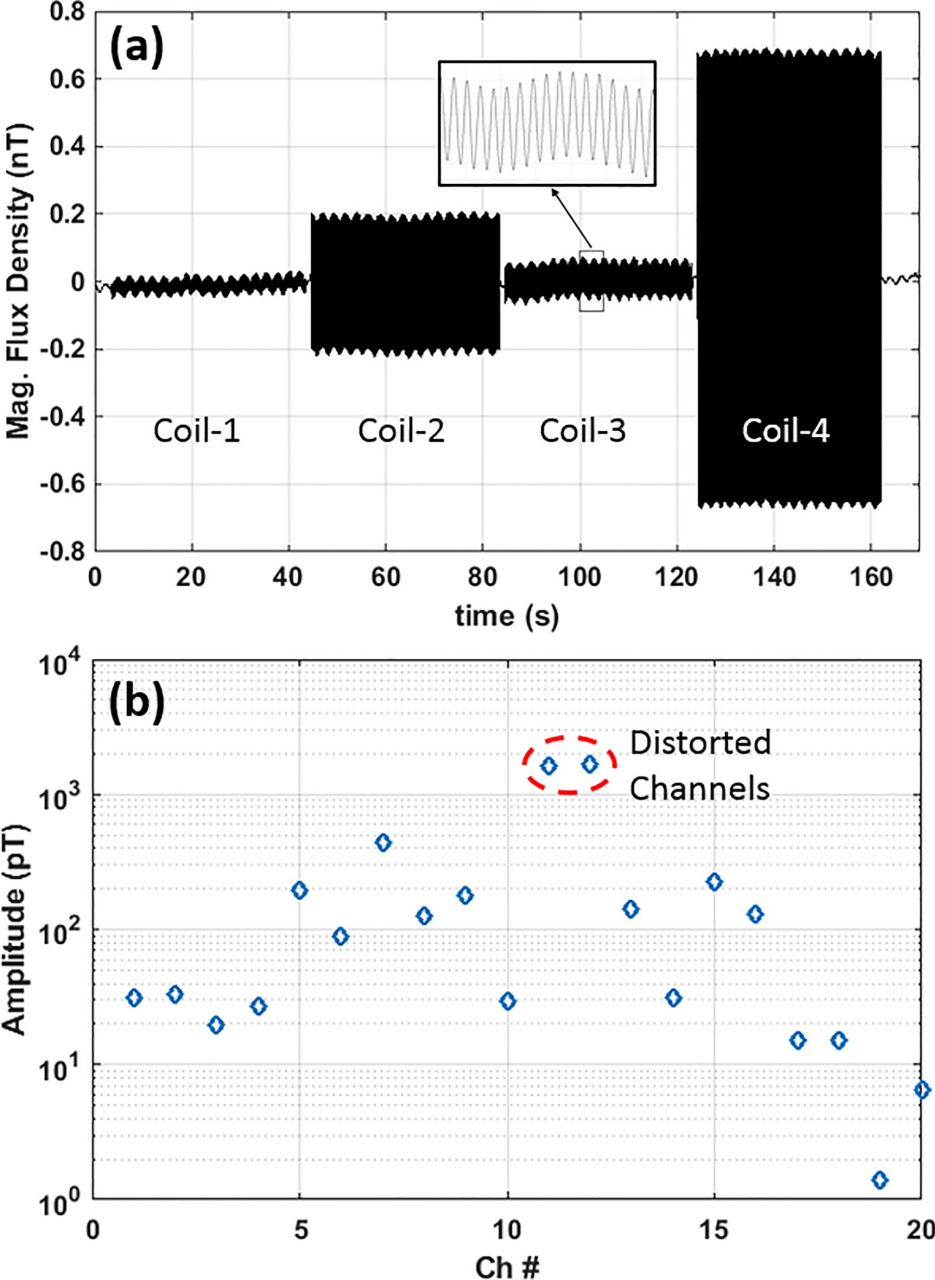
Time-domain waveforms of head position indicator (HPI) coils: (a) the raw waveform of a single channel for all the four individually activated HPI coils, and (b) channel amplitude for a single activated HPI coil.

Fig 7-A shows the continuous time-domain waveform of a single channel as it senses the magnetic fields emanating from the four HPI coils activated sequentially. After averaging the time-domain data for all cycles, channel amplitude, Fig 7-B, is generated. The linear range of our sensors is ~ 1.5 nT [8]; the magnetic field from HPI coils can exceed the upper sensing range and subsequently cause distortion in the channels located immediately adjacent to the coils. As an example, in Fig 7-B, the two channels with largest amplitude suffer from distortion. It is essential to remove these channels before localizing the position of HPI coils, as distortion is not accounted for in the forward model. We feed the time-locked data to the dipole fitting routine [27] to localize the four HPI coils. Using the calculated position of HPI coils and their digitized counterparts, we co-register the MRI and the MEG data.

**Fig 8:**
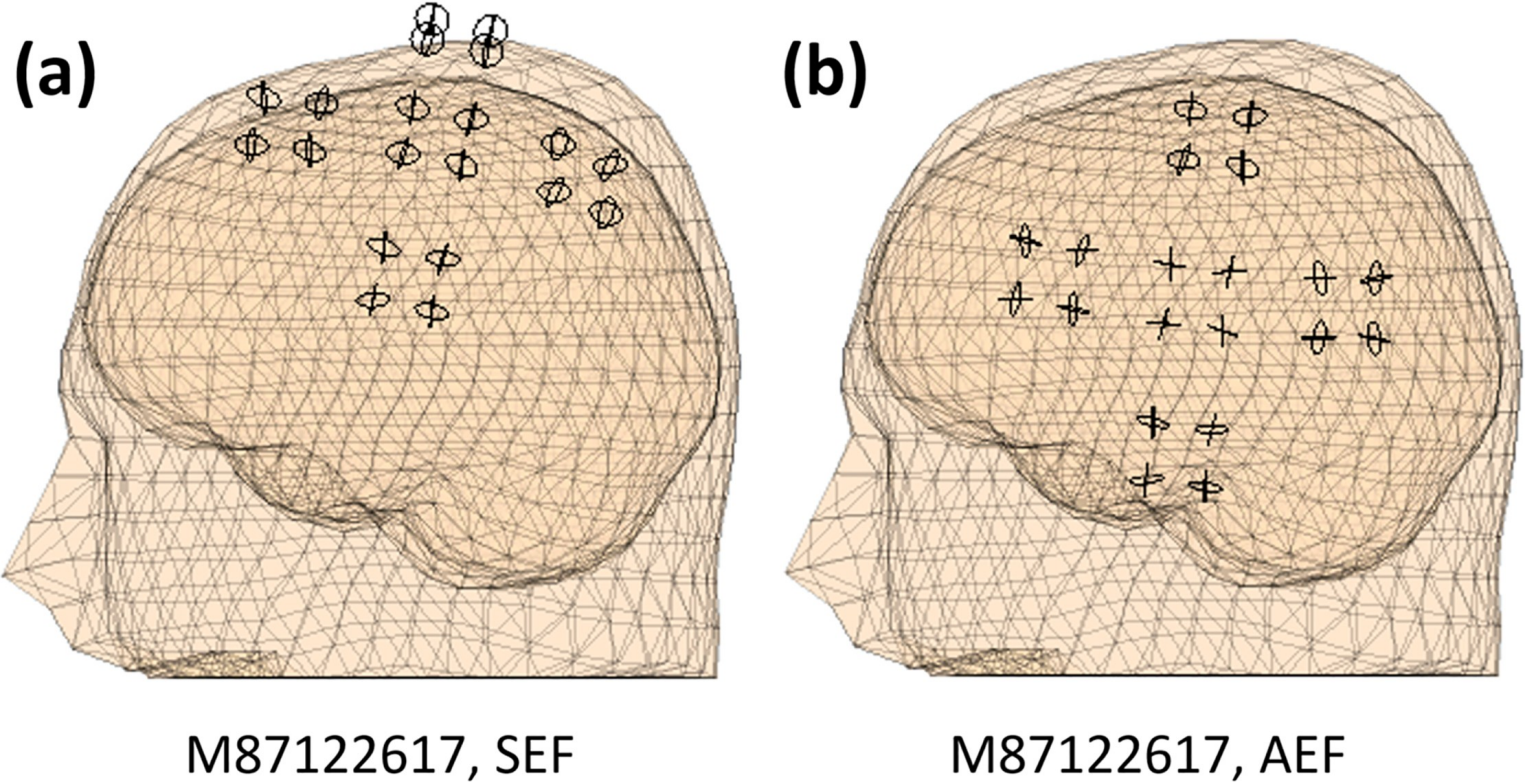
MEG-MRI co-registration. (a) SEF and (b) AEF experiments. The circles pertain to the location of OPM channels. For each MEG experiment the OPM array covers the cortex of interest.

The subjects’ magnetic resonance images are obtained using a 3-T Siemens Triotim MRI scanner providing magnetic resonance (MR) images with a spatial resolution of 1 mm. The subjects’ MR images are segmented into brain, skull, and scalp tissues using the SPM12 algorithm embedded in Fieldtrip. To achieve accurate localization, the sensors should cover the spatial magnetic field patterns generated by the targeted neuronal sources. Due to the limited number of sensors, the array could cover either the somatosensory or auditory cortex. Before each experiment the positions of the HPI coils were adjusted to cover the cortex of interest (auditory vs. somatosensory) and the subject was asked to tilt his/her head such that the targeted region of cortex was covered by the array. Fig 8 shows the location of the sensors for SEF and AEF experiments. The cortex volume, calculated by segmenting the MRI using SPM12 method embedded in the Fieldtrip software package, is used to generate a single-shell forward model [28].

### MEG signal processing

**Fig 9:**
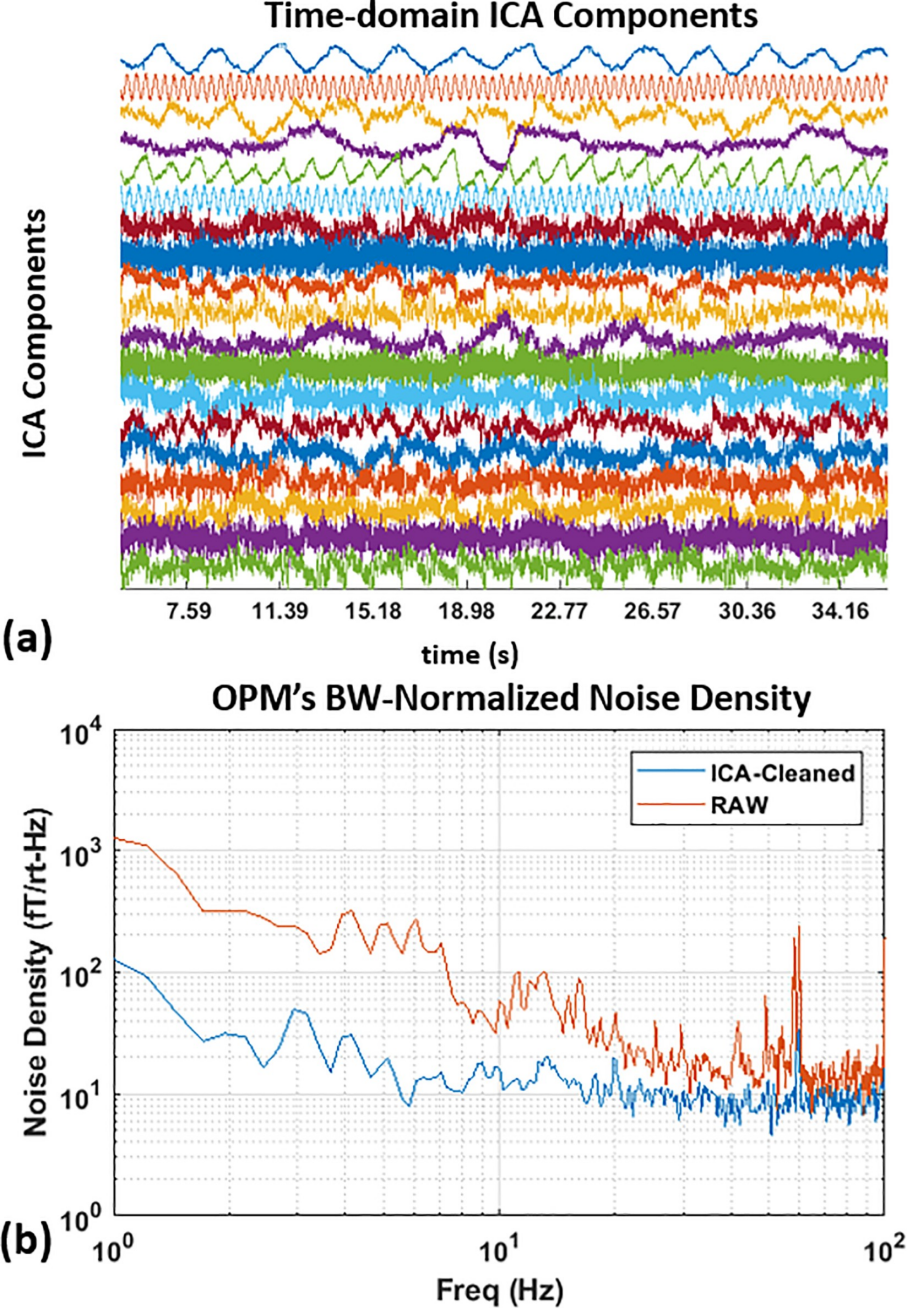
ICA components of the somatosensory evoked magnetic fields. (a) the first six components of the independent component analysis are noisy components and have no contribution to the SEF response. The MEG data will be reconstructed by removing these unwanted components entirely; and (b) noise density comparison of the raw data versus ICA-cleaned data.

Fig 9-A shows the ICA components of an SEF experiment; the first six components do not contribute to the SEF response. The noisy ICA components may have many origins such as 60 Hz, laser amplitude variation, subject’s heart beats, etc. Visual inspection of the time-locked averaged ICA components was used to manually determine whether the ICA component should be discarded or not. Fig 9-B shows improvement in noise density from cleaning the data using ICA.

**Fig 10:**
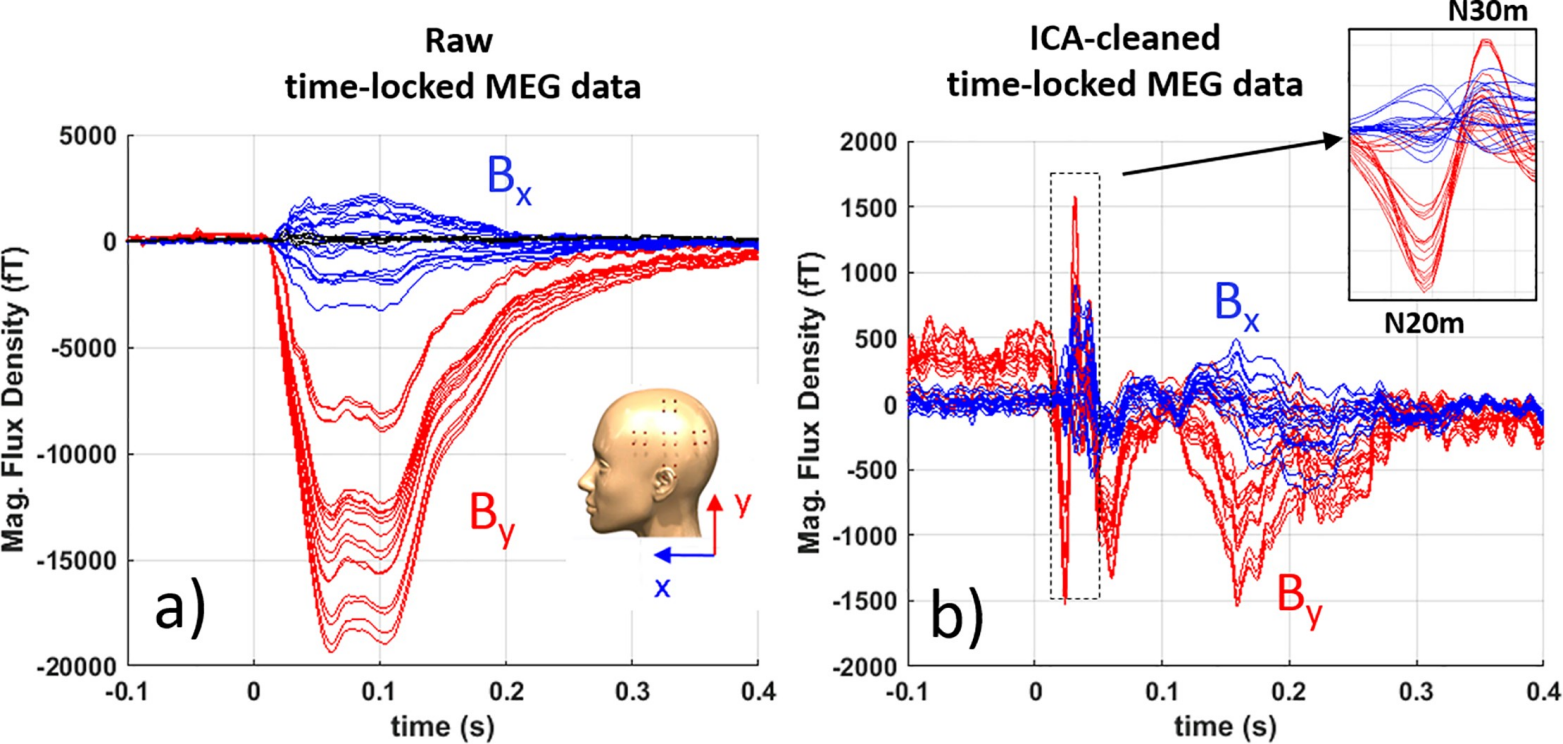
Evoked response due to median nerve stimulation showing both the horizontal and vertical field components from all 20 channels. (a) the raw data including the suspected shield artifact, and (b) the SEF data cleaned by ICA where the large 100 ms component is removed from the data. The inset in (b) depicts the N20m and N30m components. Filter: bandpass 0.1 Hz to 150 Hz.

Fig 10 shows the effect of running ICA on the SEF response; the raw data has a large component at around 100 ms which dominates the SEF’s N20m response. Using ICA, this component is successfully removed from the SEF response and in Fig 10-B N20m response is clearly visible. The large-amplitude signal is not measured by the SQUID-based MEG system, hence we speculated that its origin is not from the subject’s head. The artifact’s onset is before the N20m response leading to the conclusion that it has a different propagation path than the subject’s median nerve. We speculate that the small movement from the SEF’s thumb twitch induces movement in magnetic shield relative to the OPM array which is responsible for the up-to-20-pT-amplitude artifacts at 100 ms. In future studies, we plan to solve this issue by substantially stiffening the flat endcaps of the magnetic shield and by adding dampening materials between the shield layers.

**Fig 11:**
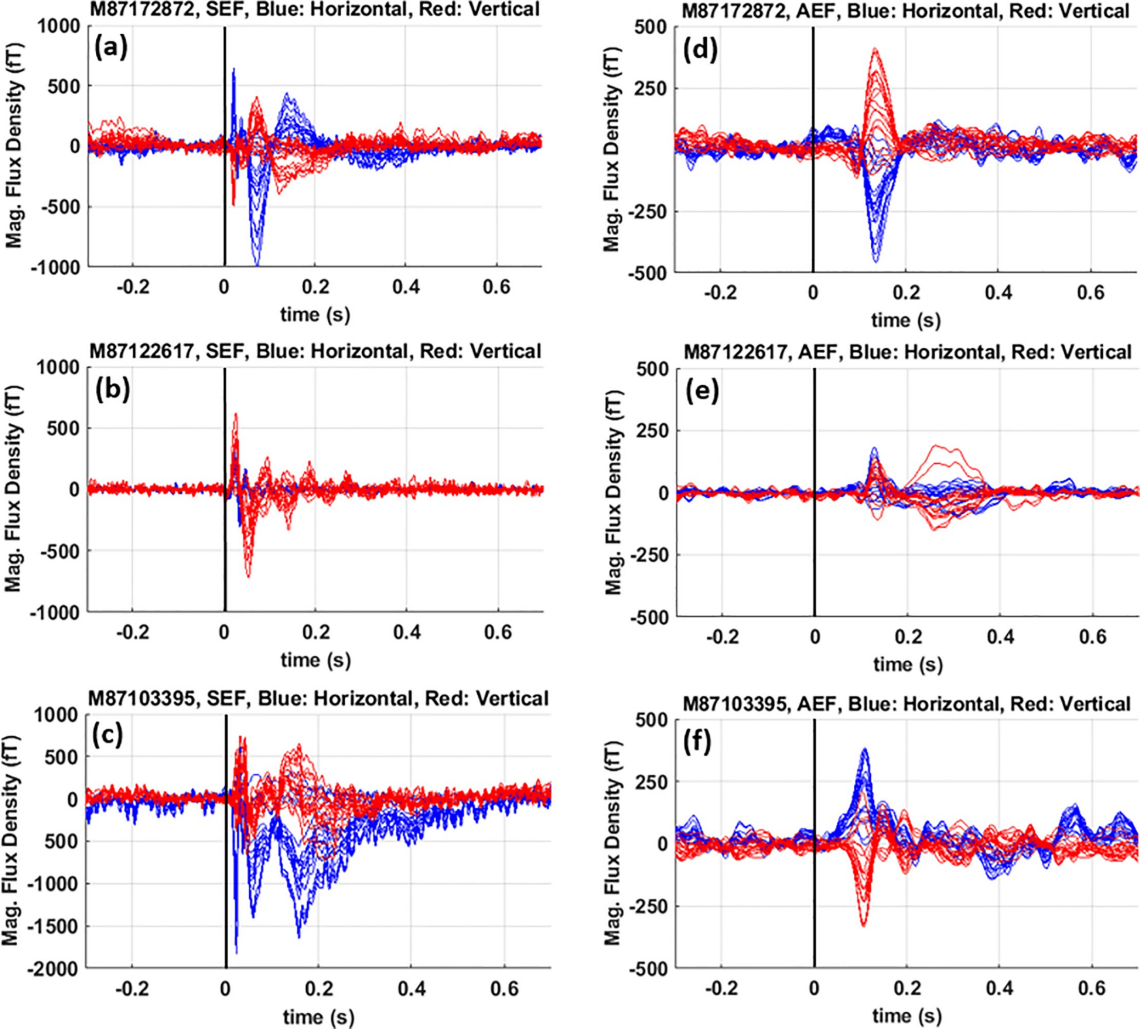
Time-domain, time-locked MEG data. (a-c) SEF and (d-f) AEF. The stimulus is presented at 0 s which its onset is indicated with the black markers.

Fig 11 shows the ICA-cleaned, time-domain, time-locked MEG data for SEF (a-c) and AEF (d-f) experiments on all three subjects. The stimulus onset is shown with a black marker.

### Measured scalp magnetic topography

**Fig 12:**
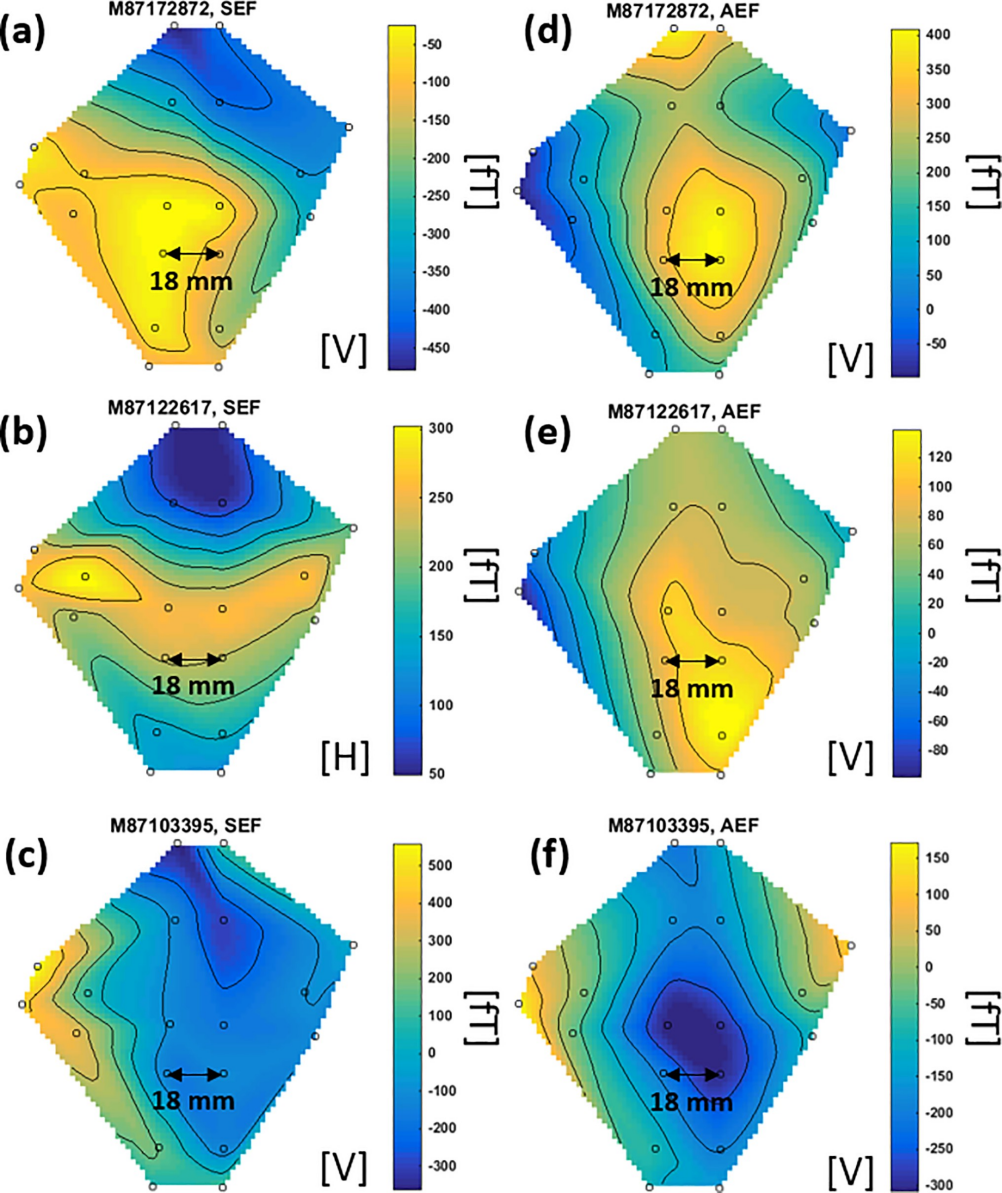
Spatial topographies of SEF (a-c) and AEF (d-f) for all three subjects. N20m and N100m peaks are used to create the field-maps for SEF (at ~20 ms latency) and AEF (at ~100 ms latency), respectively. The field-maps show the measured magnetic flux density (fT). [V]: Vertical axis measurement, [H]: Horizontal axis measurement. A cubic interpolation method is used to interpolate between channels.

The time-domain, ICA-cleaned, time-locked (averaged) MEG data (Fig 11) was used to create magnetic spatial topographies for AEF and SEF responses. It is common to create these field-maps at N20m peak (~ 20 ms) for SEF and N100m peak (~ 100 ms) for AEF data [1, 12]. Fig 12 shows the spatial topographies for SEF and AEF responses for all subjects; the small circles show the location of the OPM channels. The cubic interpolation is used to interpolate between the channels. The variation between subjects’ field-maps is due to inter-subject variations and different positions of the subjects’ heads with respect to the sensor array.

### Equivalent Current Dipole (ECD)

**Fig 13:**
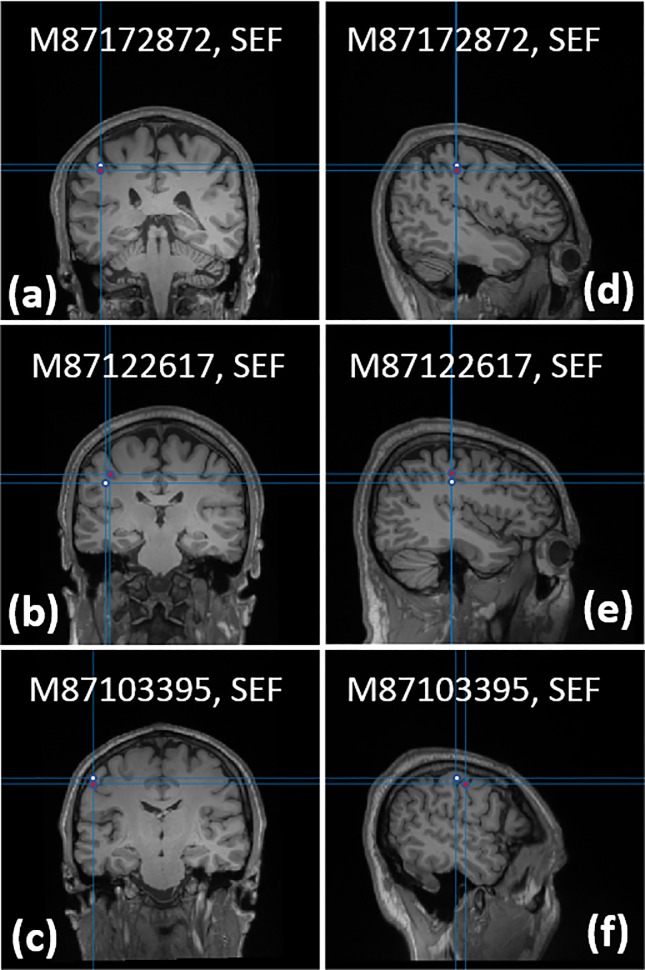
Localization of the SEF’s N20m response using equivalent current dipole fitting: (a-c) Coronal and (d-f) Sagittal planes of the dipole location. The white dot shows the dipole location yielded by the OPM-based MEG system and the red dot shows that of the commercial SQUID-based MEG system.

**Fig 14:**
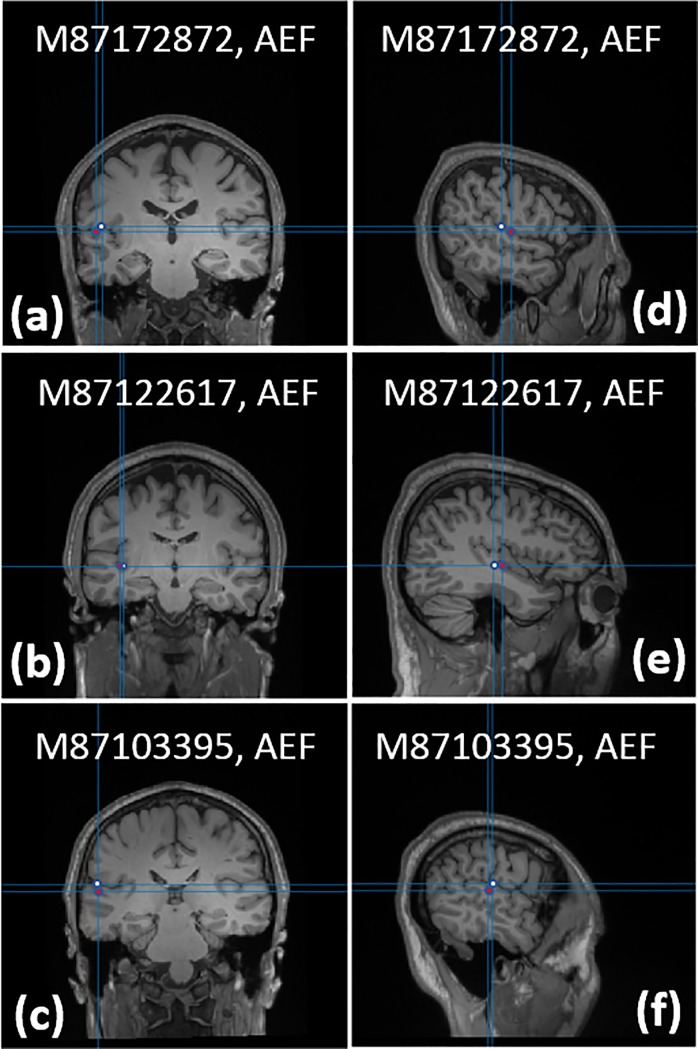
Localization of the AEF’s N100m response using equivalent current dipole fitting: (a-c) Coronal and (d-f) Sagittal planes of the dipole location. The white dot shows the dipole location yielded by the OPM-based MEG system and the red dot shows that of the commercial SQUID-based MEG system.

For each subject the SEF and AEF experiments are replicated and measured using a commercial Elekta-Neuromag SQUID-based MEG system. For the SQUID-based MEG system, the neuronal responses of SEF and AEF experiments are localized using the dipole-fitting method implemented in the Elekta-Neuromag software. Using the ICA-cleaned, time-locked SEF/AEF waveforms (Fig 11), we localized the neuronal responses for SEF/AEF experiments at N20m/N100m peaks. We used 2 ms around the response peak to average the OPM channel signal. Fig 13 shows the dipole locations calculated using the dipole-fitting routine [27] on the SEF data of all subjects collected with our OPM-based MEG system; the dipole location calculated by the SQUID-based MEG system is shown alongside that of the OPM-based MEG system. Fig 14 shows the dipole locations for the AEF response of all subjects captured using the OPM-based MEG system; the same figure contains the neuronal source location yielded by the commercial MEG system. The SQUID-based MEG data was processed using the commercial Neuromag pipeline; the commercial pipeline differs from the OPM-based MEG data processing pipeline, and the steps are as follows: 1) bandpass filter the MEG data, 2) apply temporal signal space separation method (tSSS) to remove the environmental artifacts, e.g. 60 Hz, and 3) apply signal space projection (SSP) method [29] to remove the biological artifacts, e.g. eye blink and heart beats. Table 1 compares the calculated location of the equivalent current dipole using our OPM-based MEG system to the location provided by the SQUID-based MEG system; for both SEF and AEF responses of all subjects, the error is sub-centimeter. Goodness of fit for dipole-fitting routine is shown in the OPM *rv* column which refers to the residual variance between the measured data and the reconstructed data from the localized sources. Goodness of fit for the SQUID data is stated in the column labeled SQUID gof (%) and defined in [30]. Signal to noise ratio for both OPM and SQUID data is calculated from the time-domain data and displayed in the last column of Table 1; the time-domain data before the stimulus is used to calculate the noise power and the signal power is calculated around the peak response which has inter-subject variation of a few milliseconds. Due to enhanced standoff, we expected to see an improved SNR of OPM over SQUID for shallower sources, i.e. SEF. However, we did not observe any advantage for shallower sources, i.e. SEF, over deeper sources, i.e. AEF.

**Table 1:** Comparison of the OPM- and SQUID-based MEG system performance.

Subject ID	Experiment Type	Error (mm)	OPM rv	SQUID gof (%) (26)	OPM/SQUID SNR
M87172872	SEF	3.9	0.0249	86.3	14.6/17.5
	AEF	9.5	0.0381	97.1	26.6/15.4
M87122617	SEF	7.5	0.0144	58.5	24.9/7.4
	AEF	7.1	0.0059	79.2	26.7/16.6
M87103395	SEF	9.7	0.0212	87.9	16.1/13.3
	AEF	6.2	0.0193	86.3	35.5/15.6

Ground truth is the source location provided by the commercial Elekta-Neuromag SQUID-based MEG system; the distance between the locations of the equivalent current dipole calculated by the OPM-based MEG system and that of the commercial SQUID-based MEG system is shown in the Error column. OPM *rv* refers to the residual variance between the measured data and the reconstructed data from the localized sources; goodness of fit for the SQUID data is shown in the column labeled SQUID gof (%); last column compares the signal to noise ratio of both systems.

## Analysis and discussion

To assess the localization accuracy of our OPM-based MEG system, we have used a 306-channel Elekta-Neuromag SQUID-based MEG system as our gold standard. However, both systems require separate identification of participant fiducials using a third-party device. Therefore, localization of sources in both systems will be impacted by co-registration errors. The true source may be in some other location. However, comparison to the 306-channel Elekta system allows us to determine our ability to localize commonly identified sources with similar co-registration errors. There are three main factors affecting the calculated location of the equivalent current dipole: 1) co-registration errors, 2) sensor array calibration, and 3) sensors’ signal to noise ratio. The accuracy of the forward model, i.e. lead field, depends on the volume conduction model [28], and the relative position of the subject’s head to the sensor array. In both systems, subject-specific MR images are used to construct the volume conduction model, i.e. head model. However, due to subject’s movement during the experiment, the MEG-MRI co-registration step can introduce ~ 5 mm of error [21, 31, 32]. Therefore, both SQUID and OPM array accuracy, reported in Table 1, is compromised due to errors introduced by the MEG-MRI co-registration. Calibration errors stem from errors in sensors’ sense-angle, gain, and channel locations. We have stabilized our MEG system to limit the sense-angle and gain variations to less than 1° and 5%, respectively. We have defined the OPM channel location as the geometrical center of the sensor’s sensing volume. There are three factors contributing to channel location error: 1) the sensing volume is the intersection of laser beam and the vapor cell, and its exact path is known with a ~1 mm accuracy, 2) due to absorption of the pump laser as it propagates through the vapor cell, the center of the sensing volume may be biased toward where the pump enters the vapor cell, and 3) the sensors are housed in a 3D-printed sensor array holder with an accuracy of ~1 mm. Currently, we use the channel locations provided by the coordinates of the mechanical drawings of the array holder and the sensors. Based on recent simulation studies [21], our system inaccuracy is not dominated by the calibration error. It is important to keep in mind that localization error is present in both the SQUID and OPM based systems. We chose to use the SQUID-based system as a gold-standard for a number of reasons, with a primary motivation being that the only alternative is to perform simulation studies. Despite the chance that co-registration error may occur for both systems, the fundamental physics limiting the spatial resolution of MEG applies in both cases. Strengths of the current study are that we used the exact same paradigm across the two systems as well as the same participants. Therefore, the underlying source is expected to be the same under these experimental conditions. While one sees some variability in SEF and AEF source localization across participants, the < 1-cm error within participant across different data collection days remains excellent in our opinion.

As the strength of the magnetic field decreases rapidly as a function of distance, it is obvious that reduction of the source-to-sensor distance increases the overall signal-to-noise ratio (SNR) of the signal detected by the MEG sensors. Improvements in SNR on the order of 2.7 have been reported in simulations consisting of dipolar brain sources and MEG sensors that are brought from the SQUID-related measurement distance onto the scalp [33]. In principle, however, the plain SNR value does not directly correspond to significantly improved resolution of reconstructed neural sources because nearby neural sources could still generate signal distributions detected by on-scalp MEG sensors that are highly overlapping. Thus, it could be that, e.g., two different MEG signal distributions corresponding to two nearby current dipoles would be so similar that it would be difficult to differentiate between them with certainty even when the SNR is high.

Due to the physics of MEG, however, it turns out that the inherent spatial resolution does necessarily increase with decreasing source-to-sensor distance. This is because MEG signals consist of spatiotemporal samples of quasi-static magnetic fields, which can be decomposed into components of the hierarchical vector spherical harmonic (VSH) expansion whose amplitude coefficients decay with distance at proportionally increasing rates for increasing spatial frequencies [5]. More specifically, let us denote the distances of a neural source and an arbitrary MEG sensor, measured from a point somewhere in the middle of the head, as *r’* and *r*, respectively. Then, the amplitudes of the spatial frequency components corresponding to the hierarchical multipole expansion presented in (5) decay as (*r’*/*r*)^(*l*+2)^, where *l* is the order of the expansion. Higher values of *l* correspond to higher spatial frequencies that represent higher spatial complexity of the magnetic field. Now, if we denote a typical OPM sensor distance by *r*_o_ and a corresponding SQUID sensor distance by *r*_s_, then the amplitude gain in favor of the OPM system is (*r*_s_/*r*_o_)^(*l*+2)^_._ Thus, when moving the MEG sensor array closer to the head, the amplitude contributions of high spatial frequency fields, corresponding to complex fine structure, increase proportionally more than the contributions of low spatial frequencies. Therefore, at close measurement distances the MEG signal distributions corresponding to nearby neural sources become more distinguishable than the increase in the overall SNR indicates. This fundamental resolution principle applies to all components of the three-dimensional quasistatic-magnetic field, and thus it also equally applies to the measurement of radial or transverse components of the magnetic field.

## Conclusion

We have developed a 20-channel OPM-based MEG system using our custom-designed OPM sensor. We conducted auditory evoked magnetic field (AEF) and somatosensory evoked magnetic field (SEF) experiments on three subjects. Using a commercial Elekta-Neuromag SQUID-based MEG system as a reference, the OPM-based MEG system yielded neuronal current source localization results with sub-centimeter accuracy for both AEF and SEF responses in all three subjects. Because OPMs can be placed conformally to the scalp, OPM MEG systems can lead to enhanced spatial resolution as they capture finer spatial features compared to traditional SQUID-based MEG systems.

## Supporting information

S1 File (ZIP)
